# Fitness of three chemotypes of *Fusarium graminearum* species complex in major winter wheat-producing areas of China

**DOI:** 10.1371/journal.pone.0174040

**Published:** 2017-03-17

**Authors:** Yang-yang Liu, Han-yan Sun, Wei Li, Yun-lei Xia, Yuan-yu Deng, Ai-xiang Zhang, Huai-gu Chen

**Affiliations:** 1 Institute of Plant Protection, Jiangsu Academy of Agricultural Sciences, Nanjing, China; 2 Institute of Plant Protection, Nanjing Agricultural University, Nanjing, China; Seoul National University, REPUBLIC OF KOREA

## Abstract

In China, Fusarium head blight is caused mainly by the *Fusarium graminearum* species complex (FGSC), which produces trichothecene toxins. The FGSC is divided into three chemotypes: 3-acetyldeoxynivalenol (3-ADON), 15-acetyldeoxynivalenol (15-ADON), and nivalenol (NIV). In order to predict the geographical changes in the distribution of these chemotype populations in major winter wheat-producing areas in China, the biological characteristics of twenty randomly selected isolates from each of the three chemotypes were studied. No significant difference was exhibited in the growth rate of 3-ADON, 15-ADON, and NIV isolates at 15°C. At 20°C and 25°C, the growth rate of 15-ADON isolates was the highest. At 30°C, the growth rate of NIV and 3-ADON isolates was significantly higher than that of 15-ADON isolates. The 15-ADON isolates produced the highest quantities of perithecia and two to three days earlier than the other two populations at each temperature, and released more ascospores at 18°C. The aggressiveness test on wheat seedlings and ears indicated there was no significant difference between the 3-ADON and 15-ADON isolates. However, the aggressiveness of NIV isolates was significantly lower than that of the 3-ADON and 15-ADON isolates. The DON content in grains from heads inoculated with the 3-ADON isolates was higher than the content of 15-ADON and NIV isolates. The results showed that 15-ADON population had the advantage in perithecia formation and ascospore release, and the 3-ADON population produced more DON in wheat grains. We suggested that distribution of these three chemotype populations may be related to these biological characteristics.

## Introduction

Fusarium head blight (FHB) is a major disease in wheat that is widely distributed in areas with warm and humid climate resulting in yield losses and grain quality decline [1]. In China, FHB is widely distributed and is mainly caused by members of the *Fusarium graminearum* species complex (FGSC). The FGSC can infect wheat during the period of wheat growth from seedling to ear formation stages, causing diseases such as seedling blight, crown rot, stem rot and ear rot [2]. The pathogen responsible for this disease also produces toxins that pose a threat to human and animal health [3–5]. It was found that ascospores released from perithecia on cereal crop residues were the primary source from which FHB epidemics begin [6]. In China, FHB frequently occurs in the middle and lower reaches of the Yangtze River. In recent years, due to the rise in the use of irrigation, minimum and no-tillage culture systems, and the increased density of wheat fields, FHB is gradually spreading to the northern regions of China [7].

FGSC produces trichothecene toxins and can be divided into three chemotypes: nivalenol (NIV), 15-acetyldeoxynivalenol (15-ADON), and 3-acetyldeoxynivalenol (3-ADON) [8]. Α previous investigation on the geographical distribution and composition of the phylogenetic species of the *F*. *graminearum* clade in China concluded that the 15-ADON chemotype was distributed in cooler regions with average temperatures of 15°C or lower, while the 3-ADON chemotype was more widespread in warmer regions with average temperatures above 15°C [9]. With changes in farming systems and climatic conditions, the chemotype distribution of FGSC has been altered [7]. Ward et al. [10] found that apart from its stronger reproductive capacity and growth rate, the 3-ADON population produced significantly more trichothecene than the 15-ADON population. Some studies have shown that the DON population produced more toxins than the NIV population, and the spread of the DON producing population on wheat spikes was significantly faster [11]. Moreover, experiments conducted in the Yangtze basin of China revealed that NIV isolates had a lower aggressiveness than 3-ADON isolates [12].

Analysis of the relationship between the aggressiveness and toxin production capacity of the FGSC isolates has been conducted in a number of investigations. For example, Goswami et al. [13] found that all *F*. *graminearum* isolates produced trichothecene toxins, but there was no significant correlation between aggressiveness and toxin production capacity. In contrast, Von der Ohe et al. [14] discovered that after inoculation with 3-ADON isolates, the mean FHB indexes were higher than after inoculation with 15-ADON isolates, and the average concentration of DON produced by 3-ADON isolates was significantly higher (*P* < 0.05) than that generated by 15-ADON isolates on moderately resistant and susceptible wheat lines. However, the aggressiveness and DON production of 3-ADON and 15-ADON isolates were similar in resistant lines. By identifying the species and chemotypes of 433 *Fusarium* isolates collected from wheat heads in six provinces of China, Shi et al. [15] established that there was a relationship between the aggressiveness of the species and chemotypes. Based on the analysis of the variable number of tandem repeat polymorphism (VNTR) of the Bayesian hybrid model, Zhang et al. [7] revealed that the FGSC was divided into three groups with significant genetic differentiation, and that the 15-ADON population was relatively independent. However, the genetic exchange of 3-ADON and NIV population was higher, and the distribution of the transition population was obvious. The findings of this analysis suggested that there was a substantial trend for replacement in the NIV group with the 3-ADON group.

It is speculated that the distribution of three chemotypes may be related to their adaptability to climatic conditions and the host. Thus, in this investigation, we examined the biological characteristics of 20 isolates for each chemotype and evaluated their aggressiveness in the seedling and heading stages of wheat. The aim of our study was to compare the fitness of these three chemotypes and discuss their potential for spread in China.

## Materials and methods

### Fungal isolates

Sixty isolates were randomly selected from a collection of isolates belonging to the FGSC. These samples were isolated from wheat heads with FHB symptom collected from yields in Jiangsu, Anhui, Henan, and Shandong Provinces of China in 2010, 2012, and 2013 (Table 1). The species of these isolates were identified using the partial translation elongation factor 1 alpha (*tef-*1α) gene sequences, following the method of Kristensen et al. [16]. The *Tri13* gene was used to identify chemotypes of these isolates [17, 18] (The primer sequence see S1 Table). The PCR amplification products of 3-ADON, 15-ADON and NIV chemical isolates were 644-bp, 583-bp, and 859-bp, respectively [19] (See S1 Fig). The 60 strains included the three chemotypes, and each chemotype was represented by 20 strains. Twentey strains with 15-ADON chemotype were *F*. *gramiarum*. The strains with 3-ADON and NIVchemotype were *F*. *asisticum*.

**Table 1:** Origin, species, and chemotype information for the 60 *Fusarium graminearum* Species Complex (FGSC) isolates associated with Fusarium head blight of wheat used in this study.

3-ADON			15-ADON			NIV		
Isolate	Origin	Species	Isolate	Origin	Species	Isolate	Origin	Species
F1201	Jiangsu	*F*. *asiaticum*	F1230	Anhui	*F*. *graminearum*	F1038	Jiangsu	*F*. *asiaticum*
F1203	Jiangsu	*F*. *asiaticum*	F1232	Anhui	*F*. *graminearum*	F1095	Jiangsu	*F*. *asiaticum*
F1209	Jiangsu	*F*. *asiaticum*	F1243	Henan	*F*. *graminearum*	F1096	Jiangsu	*F*. *asiaticum*
F1221	Jiangsu	*F*. *asiaticum*	F1249	Shandong	*F*. *graminearum*	F10112	Shandong	*F*. *asiaticum*
F1222	Jiangsu	*F*. *asiaticum*	F1266	Henan	*F*. *graminearum*	F10159	Jiangsu	*F*. *asiaticum*
F1226	Anhui	*F*. *asiaticum*	F1273	Shandong	*F*. *graminearum*	F1207	Jiangsu	*F*. *asiaticum*
F1233	Jiangsu	*F*. *asiaticum*	F1279	Henan	*F*. *graminearum*	F1208	Jiangsu	*F*. *asiaticum*
F1246	Anhui	*F*. *asiaticum*	F1281	Henan	*F*. *graminearum*	F1210	Jiangsu	*F*. *asiaticum*
F1252	Anhui	*F*. *asiaticum*	F1289	Shandong	*F*. *graminearum*	F1218	Jiangsu	*F*. *asiaticum*
F1301	Jiangsu	*F*. *asiaticum*	F1294	Shandong	*F*. *graminearum*	F1225	Jiangsu	*F*. *asiaticum*
F1302	Jiangsu	*F*. *asiaticum*	F1298	Henan	*F*. *graminearum*	F1227	Anhui	*F*. *asiaticum*
F1305	Jiangsu	*F*. *asiaticum*	F1307	Jiangsu	*F*. *graminearum*	F1237	Shandong	*F*. *asiaticum*
F1310	Anhui	*F*. *asiaticum*	F1315	Anhui	*F*. *graminearum*	F1257	Anhui	*F*. *asiaticum*
F1312	Anhui	*F*. *asiaticum*	F1324	Shandong	*F*. *graminearum*	F1258	Anhui	*F*. *asiaticum*
F1314	Anhui	*F*. *asiaticum*	F1325	Shandong	*F*. *graminearum*	F1259	Anhui	*F*. *asiaticum*
F1317	Anhui	*F*. *asiaticum*	F1327	Shandong	*F*. *graminearum*	F1268	Anhui	*F*. *asiaticum*
F1318	Anhui	*F*. *asiaticum*	F1330	Shandong	*F*. *graminearum*	F1285	Henan	*F*. *asiaticum*
F1320	Anhui	*F*. *asiaticum*	F1332	Shandong	*F*. *graminearum*	F1286	Henan	*F*. *asiaticum*
F1321	Anhui	*F*. *asiaticum*	F1333	Shandong	*F*. *graminearum*	F1303	Jiangsu	*F*. *asiaticum*
F1336	Jiangsu	*F*. *asiaticum*	F1334	Henan	*F*. *graminearum*	F1304	Jiangsu	*F*. *asiaticum*

### Mycelial growth rate and temperature sensitivity

The sixty isolates studied were cultured on potato dextrose agar (PDA) for 3–4 days at 25°C. Five-mm discs were taken from the edge of the colony and transferred to new 9-cm PDA plates. The plates were incubated at four different temperatures (15, 20, 25 and 30°C) in the dark for four days. On the 4^th^ day, the colony diameter was measured from two perpendicular directions, and the mean radial growth rate (millimeters per day) was determined. Each isolate was replicated three times.

### Perithecia formation and ascospore release on carrot agar

For perithecia formation, each isolate was cultured on PDA for three days at 25°C. Five-mm discs taken from the edge of the colony were positioned in the center of a 6-cm-diameter Petri dish with carrot agar. Then, the Petri dish was exposed to a black light (220–240V, 18W) and a fluorescent cool white light (18W, 6,500K) all the time at 15, 18, 21 and 25°C. The isolates were allowed to grow until mycelia reached the outer edge of the Petri dish (3–5 days). Then, the aerial mycelia were removed with a sterile inoculating needle, and 1.0 ml of 2.5% Tween 60 was added to the surface [20]. The formation of perithecia was checked 10 days after the removal of the aerial mycelia. Perithecia formation was scored as level 0 (no perithecia formed) or level 1 (1–10% of the Petri plate covered by perithecia), 2 (11–30% covered), and 3 (>30% covered) [21]. The experiment was repeated two times.

To evaluate the release of ascospores, the perithecia were formed at 18°C. On the 4^th^ day after perithecia formation, the Petri dishes were inverted. Two ml of water was dropped on the plate lids on the 6^th^, 8^th^, 10^th^, and 12^th^ day. The number of ascospores in the water was counted using a hemocytometer under a microscope (Nikon, E400, Japan). The experiment was repeated two times.

### Conidia production

Flasks containing 50 ml of 6% mung liquid medium [13, 22] were inoculated with three five-mm discs of PDA culture containing growing mycelia. The cultures were grown at 25°C on a rotary shaker at 120 rpm. After two days, the conidia concentration was quantified by a hemocytometer, and the length of 20 conidia was measured by a standalone control unit DS-L2 (Nikon). Each flask was considered as one replication and it was replicated three times.

### Seedling aggressiveness test

Kernels of Yangmai 158, a wheat cultivar that is moderately resistant to FHB, were pre-germinated in Petri dishes. Following the method described by Yang et al. [23], the germinated seedlings were nested in a plug of agar that contained growing mycelia from a 3-day-old culture on PDA. Then, 10 seedlings were wrapped and grown in a single piece of 21 × 23 cm non-woven fabrics (Shaoxing Hengsheng New Material Technology Development Co., Ltd., China) saturated with water. There were three replicates for each isolate. The fabrics were placed in a disposable cup filled with 2–3 cm of water and grown under natural light conditions. Severity was assessed on the 21^st^ day after inoculation using the modified “0–7 scale” by Li et al. [24] and Smiley et al. [25]. The following scores were used: 0, no obvious symptom; 1, light brown lesions on the coleoptile; 3, dark brown lesions on the coleoptile; 5, the 1^st^ leaf sheath becomes brown; 7, the whole plant is severely to completely necrotic. A disease index (DI) was then calculated for each replicate using the formula: DI = (∑n_s_S/7N) × 100, where S is the scale value of each plant, n_s_ is the number of plants in the category, and N is the total number of plants assessed per replicate.

### Spikelet aggressiveness test

Wheat heads of Yangmai 16 (a wheat variety moderately susceptible to FHB) were inoculated at early anthesis. Then, 10 μl of the 10^5^ spore/ml suspension was inoculated into the central spikelet [13]. Ten wheat heads were inoculated for each isolate. After inoculation, the wheat was moisted by spraying water every morning. The numbers of diseased spikelets were counted on the 7^th^, 14^th^, 21^st^, and 28^th^ day after inoculation.

### Deoxynivalenol content in wheat grains

The inoculated wheat heads were harvested, and the grains were collected with a one-ear-threshing machine. Grain was ground with a grinder (FS-II, Zhejiang Top Instrument Co. Ltd., China) and a 1.5-g portion of each ground sample was transferred into a 50-ml conical tube and extracted with 6 ml of acetonitrile-water (84:16 [vol/vol]) for four hours. Samples were then centrifuged for five minutes at 4,000 rpm. Approximately 4 ml of the sample extract was gravity-filtered through Bond Elut Mycotoxin (Agilent, USA), a newly developed solid-phase extraction sorbent that cleans up food extracts for improved trichothecene analysis. A small portion (2 ml) of the clarifying solution was absorbed and transferred into a 15-ml centrifuge tube, and was evaporated with nitrogen at 50°C. The residue in the tube was dissolved in 0.5 ml of methanol-water (20:80 [vol/vol]). Then, this was further purified with a 0.22-μm organic microporous membrane filter, and the filtrate was transferred into a vial. The amount of DON was quantified using high performance liquid chromatography (HPLC) (VARIAN, Pro Star, USA) [26].

### Analysis of trichothecenes in rice culture

The method of trichothecene production in rice culture was modified according to the studies of Burlakoti et al. [27] and Puri et al. [28]. Thirty-grams of rice grains were soaked in 100 ml of sterile distilled water in a 250 ml Erlenmeyer flask for 10 hours. Excess water was drained from the flask and was subsequently autoclaved. Three five-mm discs of agar that contained growing mycelia from a three-day-old culture on PDA were inoculated in the rice culture. The culture was then cultivated in the dark for 28 days at 22°C. For trichothecene quantification, the steps followed were in accordance with the procedure for the extraction of deoxynivalenol in wheat previously described. The content of DON and its acetylated forms (15-ADON and 3-ADON) and NIV were quantified using the liquid chromatography-mass spectrometry (HPLC–MS/MS) (QTRAP 6500, AB SCIEX, USA) method [29].

### Data analysis

Data were analyzed using SAS software Version 9 (SAS Institute, Cary, NC, USA). Conidium production and length, ascospore production, and trichothecene content were assessed using one-way analysis of variance (ANOVA). Mean mycelial growth rate was assessed at different temperatures using two-factor ANOVA analysis of variance. Because the data on aggressiveness were ordinal and not normally distributed, they were analyzed using the nonparametric method described by Shah et al. [30]. PROC RANK was used to obtain midranks, followed by PROC MIXED to calculate test statistics and significance levels.

## Results

### Mycelial growth rate and temperature sensitivity

These results revealed that the mycelial growth rate in the three chemotypes was the slowest at 15°C. With the increase in temperature, the growth rate of mycelium was accelerated and was the fastest at 25°C. However, the growth rate decreased at 30°C. The growth rate of mycelium was significantly different at four temperatures. The mycelial growth rate among the three chemotype populations was not significantly different (*P* = 0.9872), and there was an interaction between the chemotype and the temperature in the two-factor ANOVA analysis of variance. However, the mycelial growth rate of three chemotypes at some temperatures was singificantly different when the temperature factor was not taken into account. At 25°C, the growth rate of 15-ADON isolates was the highest. At 30°C, the growth rate of NIV and 3-ADON isolates was significantly higher than that of 15-ADON isolates, and the growth rate of NIV isolates was the highest (Table 2).

**Table 2:** Mycelial growth rate of the 60 *Fusarium graminearum* Species Complex (FGSC) isolates at each temperature by separated chemotype.

Chemotype	Mycelial Growth Rate (mm day^-1^)			
	15°C	20°C	25°C	30°C
3-ADON	6.98 ± 0.25 a	14.30 ± 0.56 a	15.61 ± 0.40 b	13.25 ± 0.36 b
15-ADON	6.84 ± 0.26 a	14.36 ± 0.46 a	16.03 ± 0.42 a	12.85 ± 0.38 c
NIV	6.75 ± 0.20 a	13.39 ± 0.36 b	15.24 ± 0.45 b	14.50 ± 0.33 a

Note: The values are presented as mean ± standard error. Different lowercase letters in the same column data refer to statistically significant differences at *P* < 0.05.

### Perithecia and ascospore production of the three chemotypes

**Fig 1:**
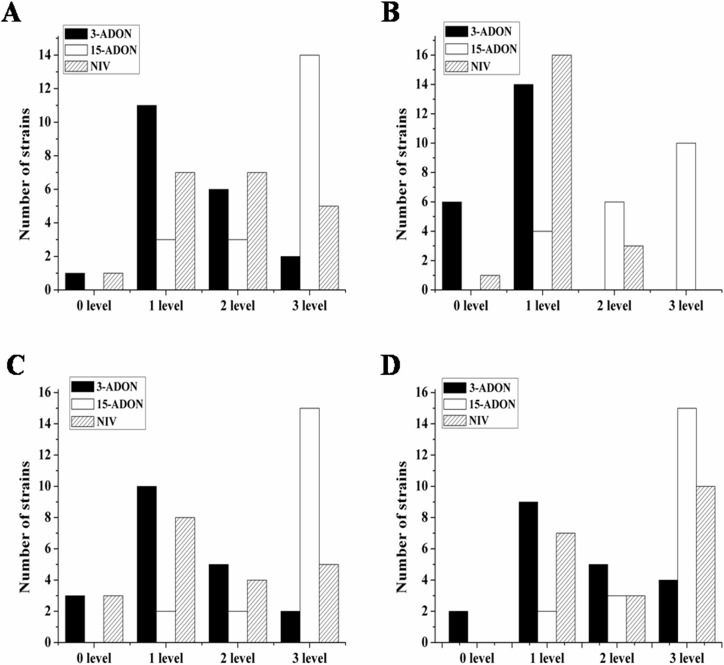
**The capacity to form the perithecia of 20 isolates of each chemotype (3-ADON, 15-ADON and NIV) at four temperatures (A, 15°C; B, 18°C; C, 21°C; D, 25°C).** Note: level 0 = 0 perithecia, level 1 = 1–10% of the Petri plate covered by perithecia, level 2 = 11–30% of the Petri plate covered by perithecia, level 3 = >30% of the Petri plate covered by perithecia.

**Fig 2:**
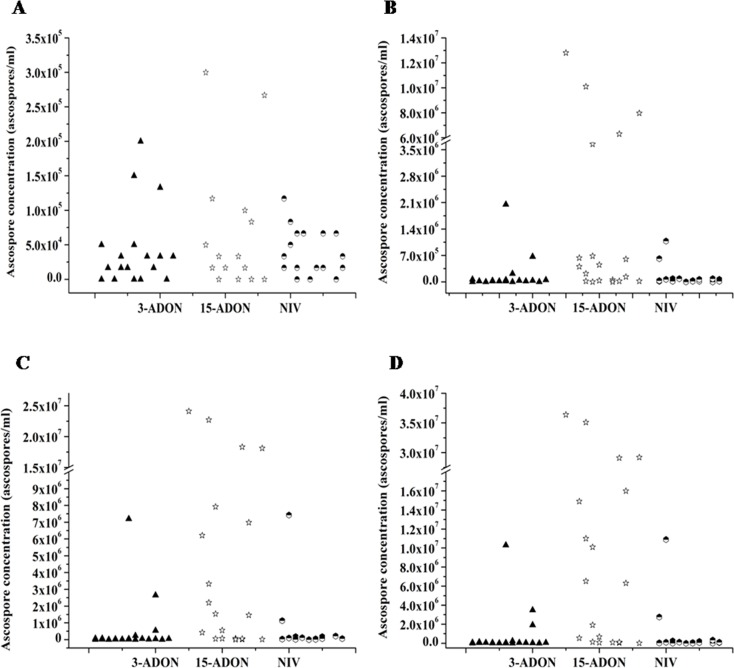
**The ascospore production of 20 isolates of each chemotype (A, 6**^**th**^**; B, 8**^**th**^**; C, 10**^**th**^**; D, 12**^**th**^ **day).** Note: The ascospores were discharged from the perithecia formed at 18°C. Each dot on the graph is represents the average concentration of total ascospores released by an isolate. The average concentration of ascospores released was the same for some isolates within a chemotype, as is reflected by less than 20 dots present in some categories.

There were substantial variations among the sixty isolates in their capacity to form perithecia, and there were inconsistencies across the temperatures for each isolate. Every tested 15-ADON isolate formed perithecia and manifested perithecia two to three days earlier than the other two chemotypes. Most of the 15-ADON isolates formed perithecia at level 3, which indicated that the perithecia covered >30% of the Petri dish. Meanwhile, some 3-ADON and NIV isolates did not form perithecia at some temperatures, and most of the isolates formed perithecia at level 1 (Fig 1). In the ascospore release experiment, the majority of isolates began to release ascospores on the 4^th^ day. On the 6^th^ day, the ascospore release of the three chemotype isolates was similar. On the 8^th^ day, 15-ADON isolates released more ascospore than 3-ADON and NIV isolates. Up to the 12^th^ day, the average concentration of ascospores released by 15-ADON isolates was 4.20 × 10^6^/ml, which was about ten times the quantity released by the 3-ADON (2.74 × 10^5^ ascospore/ml) and NIV isolates (2.70 × 10^5^ ascospore/ml) (Fig 2).

### Conidia production

Results revealed that the macroconidia production of 15-ADON isolates was the highest with a concentration of 5.41 × 10^5^ macroconidia/ml. The potential for the 3-ADON and NIV isolates to produce conidia were similar. Furthermore, the 15-ADON isolates had the longest macroconidia, which was significantly longer than those generated by the 3-ADON and the NIV isolates. However, there was no significant difference in macroconidia production between the 3-ADON and 15-ADON isolates (Table 3).

**Table 3:** The conidium production and length of the 60 *Fusarium graminearum* Species Complex (FGSC) isolates by separated chemotype.

Variable	Chemotype		
	3-ADON	15-ADON	NIV
Concentration (10^5^/ml)	3.86 ± 0.45 ab	5.41 ± 0.50 a	2.68 ± 0.31 b
Conidium length (μm)	44.93 ± 0.79 b	59.25 ± 1.02 a	44.85 ± 0.78 b

Note: The values are presented as mean ± standard error. Different lowercase letters in the same row data refer to statistically significant difference at *P* < 0.05.

### Aggressiveness on wheat

First, aggressiveness of the isolates at the wheat seeding stage was tested. The results indicated that on the 14^th^ day after inoculation, the symptoms began to appear on wheat seeding bases. On the 21^th^ day after inoculation, the average disease index of 3-ADON, 15-ADON and NIV were 72.71, 70.72 and 48.27, respectively. Variance analysis revealed that there was no significant difference between 3-ADON and 15-ADON isolates for disease index, but the average disease indexes of the NIV chemotype isolates were significantly lower than that of the 3-ADON and 15-ADON isolates (Table 4). On wheat heads, more diseased spikelets were observed in the 3-ADON isolates (Table 4).

**Table 4:** Aggressiveness of the 60 *Fusarium graminearum* Species Complex (FGSC) isolates by separated chemotype.

Days	Chemotype		
	3-ADON	15-ADON	NIV
Average number of spikelets infected			
7 d	0.67 ± 0.17 a	0.5 ± 0.14 b	0.31 ± 0.06 c
14 d	2.57 ± 0.92 a	2.33 ± 0.70 a	0.67 ± 0.29 b
21 d	11.44 ± 3.35 a	9.14 ± 2.76 b	2.40 ± 1.57 c
28 d	16.43 ± 2.41 a	15.00 ± 2.12 a	8.22 ± 2.73 b
Average disease index on seedling			
21d	72.71 ± 0.03 a	70.72 ± 0.04 a	48.27 ± 0.03 b

Notes: Average disease index on seedling was assessed on the 21^st^ day after inoculation. The values are expressed as mean ± standard error. Different lowercase letters in the same row data refer to statistically significant differences at *P* < 0.05.

### Trichothecene production

All isolates generated DON in grains at levels ranging from 0.15 to 36.78 μg/g. DON was the main trichothecene produced by all isolates though the concentration varied among isolates of the same chemotype group. The 3-ADON isolates produced more DON than the 15-ADON and NIV isolates. Variance analysis revealed a significant difference in the DON content among three chemotypes populations (Table 5). The trichothecene production by the 60 isolates in the rice culture were also evaluated. The 3-ADON and 15-ADON isolates produced mainly DON, while the NIV isolates formed primarily NIV. The DON concentration ranged from 0.04 to 93.66 μg/g, and the NIV concentration ranged from 0.04 to 65.23 μg/g. Two 15-ADON isolates (F1327 and F1334) manifested the highest DON production. The mean DON content produced by the 15-ADON isolates was the highest and was significantly higher than that produced by 3-ADON and NIV in rice culture (Table 5).

**Table 5:** Trichothecene production of the 60 *Fusarium graminearum* Species Complex (FGSC) isolates in rice culture and spikelets by separated chemotype.

Toxin content (μg/g)	Chemotype		
	3-ADON	15-ADON	NIV
Toxin in rice culture			
DON	31.28 ± 3.38 b	48.29 ± 5.15 a	0.71 ± 0.15 c
3-ADON	4.01 ± 0.55 a	0.36 ± 0.08 b	0.09 ± 0.04 b
15-ADON	7.50 ± 1.62 a	1.60 ± 0.53 b	0.18 ± 0.03 b
NIV	0.46 ± 0.10 b	0.40 ± 0.10 b	16.25 ± 3.77 a
Toxin in wheat spikelet			
DON	15.00 ± 1.86 a	8.38 ± 3.39 b	0.47 ± 0.09 c

Note: The values are presented as mean ± standard error. Different lowercase letters in the same row data refer to statistically significant differences at *P* < 0.05.

## Discussion

The primary pathogen of FHB in China was identified as *F*. *graminearum* (teleomorph *Gibberella zeae*) until O’Donnell et al. [31] used genealogical concordance phylogenetic species recognition (GCPSR) to investigate species limits in *F*. *graminearum*. Since then, some reports have showed that *F*. *graminearum* of China falls into *F*. *asiaticum* and *F*. *graminearum* sensu stricto. *F*. *asiaticum* was the dominated species in the middle and lower reaches of the Yangtze River and *F*. *graminearum* is more common in northern China. 3-ADON and NIV were the main chemotypes in *F*. *asiaticum*. In *F*. *graminearum*, only 15-ADON chemotype was found [32– 34]. Based on VNTR data and Bayesian model, Zhang et al. [7] found that FGSC could be divided into three populations, which were 3-ADON, 15-ADON and NIV.

Zhang et al. [7] also revealed that the 3-ADON population from China had significant advantages in seven biological characteristics, and suggested that the 3-ADON population might replace the NIV population. The current study also showed that the NIV population was less virulent and produced lower amounts of trichothecene than the 3-ADON population, but NIV population was similar to 3-ADON in perithecia forming, ascospore release and conidium production. NIV and 3-ADON population differentiated on the mycelial growth at tested temperatures although both chemotypes are *F*. *asiaticum*. NIV population appears more adapt to high temperature. The growth rate of the NIV population was lower at 15°C and significantly higher than that of 3-ADON and 15-ADON populations at 30°C. Moreover, in recent years, we have recovered stable proportions of NIV producing isolates [33, 35], but found no indication that the NIV population was replacing the 3-ADON population. That may be related to the higher temperature adaption of NIV population.

In this study, we unexpectedly found that more perithecia were formed at an earlier period by the 15-ADON chemotype isolates. The 15-ADON chemotype population also released more ascospores than the other two chemotype populations. Spolti et al. [21] compared some traits of 15-ADON and 3-ADON isolates from New York, and found that isolates of two chemotypes could not differentiated for most of tested traits. They also found that 15-ADON isolates were faster and more abundant producers of perithecia on carrot agar, but the 3-ADON isolates produced two times more ascospores than 15-ADON isolates. Fuentes-Bueno [36] proposed that errors in the measurements of ascospore production might have been caused by differences in the duration of perithecia maturation. Thus, we performed four-fold measurements. Some previous examinations investigated the ascospore discharge under conditions with high moisture or in humidity chambers [20, 37, 38]. During our study, we applied plate lids to collect and quantify the released ascospores and found the method is easily applicable and can eliminate the disadvantage of the uneven distribution of perithecia on carrot agar.

It has been well-accepted that the 15-ADON chemotype isolates predominate the FHB pathogen population in northern China, and the 3-ADON and NIV isolates are found mainly in the south. Based on results of this study, the advantage on perithecia formation and ascospore release may be critical for the 15-ADON population in the north, because the conditions for perithecia formation there such as temperature and moisture on the soil surface are less favorable than that in the south. In a previous study, 3-ADON isolates were found in the crown rot pathogen population even in the far north [35]. This finding suggests that the 3-ADON population exists in soil in northern China, and infects wheat crown with mycelia, but the 15-ADON isolates is predominant in the FHB pathogen population due to better perithecia formation and ascospore release capability. In contrast, most wheat varieties in the south have greater resistance to FHB, and the quantity of ascospores is sufficient to cause an FHB epidemic [6]. Aggressiveness and trichothecene production may be more important for the 3-ADON population to cope with host resistance and compete with other microbes.

Although the capability of the 3-ADON population for perithecia formation and ascospore release was lower than that of the 15-ADON population, its aggressiveness and DON production in wheat were higher. With changes in global climate change and increasing temperatures, the conditions for perithecia formation are improving. Whether the 3-ADON population will move towards the northern regions of China and replace the 15-ADON population is worth monitoring.

## Supporting information

S1 Fig PCR amplification of chemotypes of the *F*. *graminearum* species complex isolates. PCR amplification of chemotypes of 15-ADON (583 bp), 3-ADON (644 bp) and NIV (859 bp). M: 100 bp DNA ladder, Lanes 1–17 were *F*. *graminearum* species complex isolates.
(TIF)

S1 Table Primer used in this study. (DOCX)
