# Supplementary material for: Fitness of three chemotypes of Fusarium graminearum species complex in major winter wheat-producing areas of China
Source: PLoS One. 2017 Mar 17;12(3):e0174040. doi: 10.1371/journal.pone.0174040 (PMC5357014; doi:10.1371/journal.pone.0174040)
Supplement: S1 Table — (DOCX) [file pone.0174040.s002.docx]

**S1 Table. Primer used in this study.**

| Primer | Nucleotide sequence（5’-3’） | Reference |
| --- | --- | --- |
| EF1 | ATGGGTAAGGAAGACAAGAC | Kristensen et al. [16] |
| EF2 | GGAAGTACCAGTGATCATGTT | Kristensen et al. [16] |
| Tri13P1 | CTCSACCGCATCGAAGASTCTC | Wang et al. [18] |
| Tri13P2 | GAASGTCGCARGACCTTGTTTC | Wang et al. [18] |
